# Cognitive control of orofacial motor and vocal responses in the ventrolateral and dorsomedial human frontal cortex

**DOI:** 10.1073/pnas.1916459117

**Published:** 2020-02-14

**Authors:** Kep Kee Loh, Emmanuel Procyk, Rémi Neveu, Franck Lamberton, William D. Hopkins, Michael Petrides, Céline Amiez

**Affiliations:** ^a^Univ Lyon, Université Lyon 1, INSERM, Stem Cell and Brain Research Institute U1208, 69500 Bron, France;; ^b^Institut de Neurosciences de la Timone, Aix-Marseille Université, CNRS, UMR 7289, 13005 Marseille, France;; ^c^Groupe d’Analyse et de Théorie Economique, CNRS UMR 5229, Université de Lyon, 69003 Lyon, France;; ^d^La Structure Fédérative de Recherche Santé Lyon-Est, CNRS UMR 3453, INSERM US7, Lyon 1 University, 69008 Lyon, France;; ^e^Centre d’Etude et de Recherche Multimodal et Pluridisciplinaire en Imagerie du Vivant (CERMEP), 69677 Bron, France;; ^f^Department of Comparative Medicine, Keeling Center for Comparative Medicine and Research, The University of Texas MD Anderson Cancer Center, Bastrop, TX 78602;; ^g^Department of Neurology and Neurosurgery, Montreal Neurological Institute, McGill University, Montreal, QC H3A 2B4, Canada;; ^h^Department of Psychology, McGill University, Montreal, QC H3A 1G1, Canada

**Keywords:** speech evolution, vocal control, supplementary motor cortex, Broca’s area, midcingulate cortex

## Abstract

In the primate brain, a set of areas in the ventrolateral frontal (VLF) cortex and the dorsomedial frontal (DMF) cortex appear to control vocalizations. The basic role of this network in the human brain and how it may have evolved to enable complex speech remain unknown. In the present functional neuroimaging study of the human brain, a multidomain protocol was utilized to investigate the roles of the various areas that comprise the VLF–DMF network in learning rule-based cognitive selections between different types of motor actions: manual, orofacial, nonspeech vocal, and speech vocal actions. Ventrolateral area 44 (a key component of the Broca’s language production region in the human brain) is involved in the cognitive selection of orofacial, as well as, speech and nonspeech vocal responses; and the midcingulate cortex is involved in the analysis of speech and nonspeech vocal feedback driving adaptation of these responses. By contrast, the cognitive selection of speech vocal information requires this former network and the additional recruitment of area 45 and the presupplementary motor area. We propose that the basic function expressed by the VLF–DMF network is to exert cognitive control of orofacial and vocal acts and, in the language dominant hemisphere of the human brain, has been adapted to serve higher speech function. These results pave the way to understand the potential changes that could have occurred in this network across primate evolution to enable speech production.

## Significance

Across primates, a set of ventrolateral frontal (VLF) and dorsomedial frontal (DMF) brain areas are critical for voluntary vocalizations. Determining their individual roles in vocal control and how they might have changed is crucial to understanding how the complex vocal control in human speech emerged during primate brain evolution. The present work demonstrated key functional dissociations in Broca’s region of the VLF (i.e., between dorsal and ventral area 44, and area 45) and in the DMF (i.e., between the presupplementary motor area [pre-SMA] and the midcingulate cortex [MCC]) during the cognitive control of orofacial, nonspeech, and speech vocal responses.

The question of how the complex vocal control underlying human speech and its neural correlates emerged during primate evolution has remained controversial because of the difficulty in accepting continuity between highly flexible human speech and nonhuman primate (NHP) vocalizations, which appear to be limited to a set of fixed calls that are tied to specific emotional and motivational situations (1). However, recent evidence is suggesting that volitional and flexible vocal control is indeed present in NHPs (2). Furthermore, the complexity of cognitive vocal control appears to increase across the primate phylogeny: although monkeys can flexibly initiate and switch between innate calls (3, 4), chimpanzees and orangutans are capable of acquiring species-atypical vocalizations and using them in a goal-directed manner (5, 6). Importantly, the cytoarchitectonic homologs of Broca’s speech region (i.e., areas 44 and 45) in the human brain have been recently established in the NHP brain (7, 8). Thus, human speech, and its neural correlates, could have evolved from a basic cognitive vocal control system that already exists in NHPs (2). The identification of this early cognitive vocal control system and its generic functions would be a critical step forward in understanding the emergence of human speech during primate evolution.

Across primates, two anatomically homologous frontal systems are implicated in the cognitive control of vocalizations (2): (i) the ventrolateral frontal cortex (VLF) that includes cytoarchitectonic areas 44 and 45, and which, in the language dominant hemisphere of the human brain, is referred to as Broca’s region; and (ii) the dorsomedial frontal cortex (DMF), which includes the midcingulate cortex (MCC) (9), as well as the immediately dorsal supplementary motor area (SMA) and the presupplementary motor area (pre-SMA) (10, 11). In the language-dominant hemisphere of the human brain, damage to the VLF region yields severe speech impairments (12). Electrical stimulation of the cortex on the ventrolateral frontal cortex, which lies immediately anterior to the ventral precentral premotor/motor cortex that is involved in the control of the orofacial musculature, results in pure speech arrest (13). The ventrolateral frontal cortex immediately anterior to the ventral premotor cortex yielding speech arrest is the pars opercularis, where area 44 is located. Anterior to this region lies area 45 on the pars triangularis. Functional neuroimaging studies suggest a role of area 45 in the active controlled verbal memory retrieval (14), which is often expressed in verbal fluency (15, 16). Consistent with this earlier work, Katzev and colleagues (17) demonstrated a dissociation between areas 44 and 45 in verbal production: area 44 was more active during the phonological retrieval of words and area 45 during the controlled semantic retrieval of words. Electrical stimulation in the DMF region of the human brain results in vocalization in a silent patient and speech interference or arrest in a speaking patient (18), and DMF lesions have been associated with long-term reduction in verbal output. Importantly, Chapados and Petrides (19) noted that DMF lesions must include SMA, pre-SMA, and the MCC regions to induce deficits, suggesting the existence of a local DMF network contributing to vocal and speech production.

**Fig. 1.**
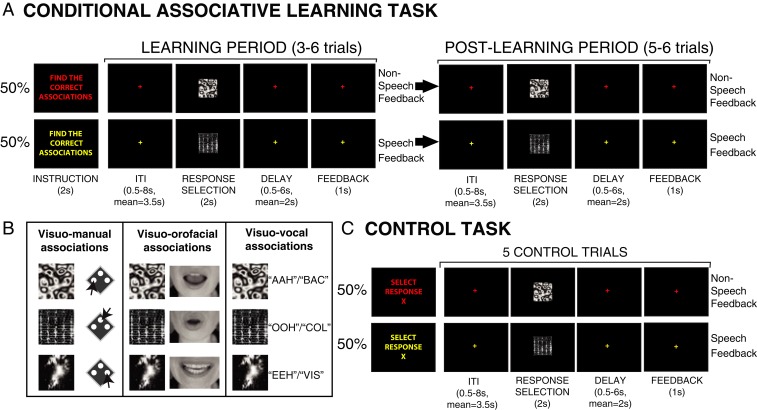
Experimental tasks. (*A*) Conditional associative learning task. In the learning phase, subjects have to discover the correct pairings between three motor responses and visual stimuli. On each trial, one visual stimulus was randomly presented for 2 s, to which the subjects selected one of three motor responses (response selection). If the instruction font and fixation cross were red (in 50% of learning sets), nonspeech feedback (FB) was provided to indicate whether the response was correct (“AHA”) or wrong (“BOO”). If the instruction font and fixation cross were yellow, speech feedback was provided to indicate whether the response was correct (“CORRECT”) or wrong (“ERROR”). After a correct response was performed to the three stimuli (marking the end of the learning period), the subjects had to perform each of the learned associations twice (i.e., postlearning period). (*B*) Visuo-motor associations in the three versions of conditional associative learning task. In the visuo-manual condition, subjects learn associations between three button presses and three visual stimuli. In the visuo-orofacial condition, subjects learn associations between three orofacial movements and three visual stimuli. In the visuo-vocal condition, subjects learn associations between three nonspeech vocalizations (“AAH,” “OOH,” “EEH”) or three speech vocalizations (“BAC,” “COL,” “VIS”; the French words that respectively refer to “trough,” “collar,” and “screw” in English) and three visual stimuli. (*C*) Visuo-motor control task with nonspeech (50% of trials, indicated by red fonts and fixation crosses) or speech feedback (50% of trials, indicated by yellow fonts and fixation crosses). In the control task, the subjects perform the instructed (X) motor response to every presented stimulus during response selection for five consecutive trials.

The present functional neuroimaging study of the human brain seeks to disentangle the individual roles of the various VLF–DMF areas in cognitive vocal control that might be generic across primates. In both the human and the macaque brains, the posterior lateral frontal cortex that lies immediately anterior to the precentral motor zone has been linked to the cognitive selection between competing motor acts (20), and MCC has been typically associated with behavioral feedback evaluation during learning (21). On this basis, we hypothesize that area 44 is involved in the high-level cognitive selection between competing orofacial and vocal acts, while the MCC is involved in the use of vocal feedback for adapting vocal behaviors. To evaluate these hypotheses, we utilized a multidomain conditional associative learning and performance response protocol (20, 22, 23). The protocol requires subjects to select between competing acts based on learned conditional relations (i.e., if stimulus A is presented, then select response X, but if stimulus B is presented, then select response Y, etc.). The subjects must, therefore, first learn by trial and error to select one from a set of particular responses based on these if/then relations (learning period). During this learning period, nonspeech and speech feedback is provided. Once the correct associations are learned, subjects must repeat them (postlearning period; Fig. 1*A*). During both periods, we examined functional activations in the VLF–DMF network during the selection of (i) orofacial, (ii) nonspeech vocal, (iii) speech vocal, and (iv) manual acts, as well as during the processing of (i) nonspeech vocal and (ii) speech vocal feedback (Fig. 1*B*).

The results provided major insights into the contributions of the various VLF–DMF areas in orofacial, nonspeech vocal, and speech vocal production. In the VLF network, area 44 is involved in the cognitive selection of orofacial as well as both nonspeech and speech vocal responses (but not manual responses) based on conditional learned relations to external stimuli (i.e., cognitive rule-based selection between competing alternative responses). In contrast, area 45 is specifically recruited during the selection of both nonspeech and speech vocal responses but only during learning, i.e., when the if/then conditional relations have not yet been mastered and, therefore, the active cognitive mnemonic retrieval load is high. In the DMF network, the MCC is involved in processing auditory nonspeech and speech vocal feedback and effector-independent cognitive response selection during learning, but the pre-SMA is only involved in the cognitive control of speech vocal response selections based on speech vocal feedback.

## Results

The subjects underwent three functional magnetic resonance imaging (fMRI) sessions during which they performed a visuo-motor conditional associative learning task (Fig. 1*A*) and the appropriate control task (Fig. 1*C*) with different motor responses (Fig. 1*B*): orofacial acts (mouth movements), vocal acts, i.e., both nonspeech and speech vocalizations, and, as a control, manual acts (button presses). In each learning task block (Fig. 1*A*), the subjects first learned the correct conditional relations between three different visual instructional stimuli and motor responses (if stimulus A, select response X, but if stimulus B, select response Y, etc.) based on the nonspeech vocal or speech vocal feedback provided (learning phase), and subsequently executed the learned associations (postlearning phase). In each control task block (Fig. 1*C*), the subjects performed an instructed response to three possible visual stimuli, i.e., the visual and motor aspects of the task were identical to those in the conditional selection task, but, critically, no cognitive selection based on prelearned cognitive if/then rules or feedback-driven adaptation were required in the control task.

### Functional Dissociations in the Posterior Lateral Frontal Cortex (Dorsal Premotor Region and Ventral Area 44) during Cognitive Manual, Orofacial, and Vocal Selections.

**Fig. 2.**
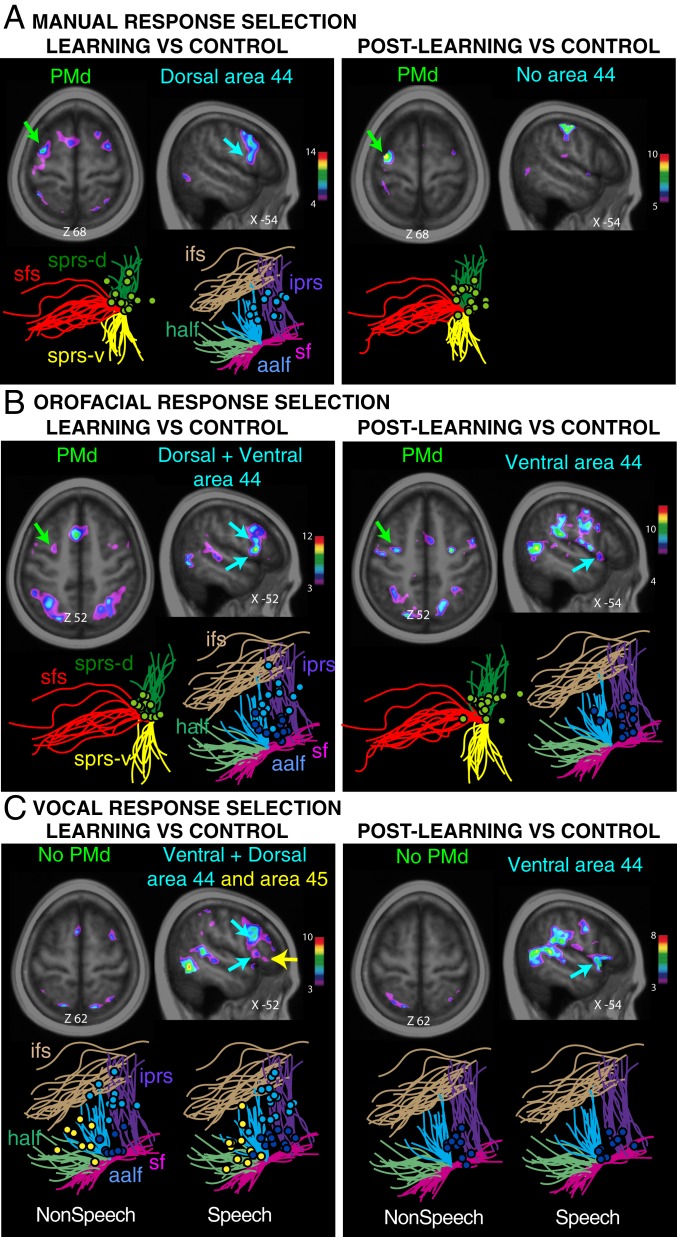
Functional dissociations in the posterior lateral frontal cortex during cognitive manual, orofacial, and vocal response selections. Group (above) and individual subject activations (below; shown as dots around relevant sulci) during response selection in learning and postlearning periods relative to control for (*A*) manual, (*B*) orofacial, and (*C*) nonspeech and speech responses. Green circles depict individual PMd activations. Light and dark blue circles depict individual dorsal and ventral area 44 activations. Yellow circles depict area 45 activations. Abbreviations: aalf, anterior ascending ramus of the lateral fissure; cs, central sulcus; half, horizontal ascending ramus of the lateral (Sylvian) fissure; ifs, inferior frontal sulcus; iprs, inferior precentral sulcus; sf, Sylvian (lateral) fissure; sfs, superior frontal sulcus; sprs-d, dorsal superior precentral sulcus; sprs-v, ventral superior precentral sulcus.

The BOLD signal during response selection was examined between postlearning versus control trials and between learning versus control trials involving manual, orofacial, and nonspeech and speech vocal responses. In line with previous findings (22), group-level analyses demonstrated increased activity in the left dorsal premotor region (PMd) during the conditional selection of manual responses, in both the learning and postlearning trials, relative to the appropriate control trials (Fig. 2*A*; *SI Appendix*, Table S1 shows activation peak locations and *t*-values). Single-subject analyses confirmed that individual left PMd peaks, during both the learning (observed in 17 of 18 subjects) and postlearning phases (observed in 18 of 18 subjects) of manual responses, were consistently located in the dorsal branch of the superior precentral sulcus, as previously demonstrated (22, 24). Importantly, no significant activation was observed in the pars opercularis, i.e., the part of the ventrolateral frontal cortex where area 44 lies, during the postlearning periods of conditional manual selections. During the learning period, activation was observed in the dorsal part of area 44 (Fig. 2*A* and *SI Appendix*, Table S1). Thus, the results of the manual task replicate the known role of the dorsal premotor region in the cognitive selection of manual acts (20, 22, 24) and provide the background necessary to ask questions about the role of area 44 in the orofacial and vocal (speech and nonspeech) acts.

By contrast to manual response selection, orofacial response selection resulted in increased BOLD activity in both the left ventral area 44 and PMd during the learning and postlearning periods (Fig. 2*B*; *SI Appendix*, Table S1 shows activation peak locations and *t*-values). Subject-level analyses showed that the individual left ventral area 44 peaks were observed in the pars opercularis (14 of 18 subjects in both the learning and postlearning periods), and the left PMd peaks were consistently found in the dorsal branch of the superior precentral sulcus (observed in 14 of 18 subjects during the learning period and in 17 of 18 subjects in the postlearning period; Fig. 2*B*).

The cognitive selection of vocal responses (pooled across nonspeech and speech vocal responses) was associated with increased BOLD activity in the left ventral area 44, and not the PMd, during both the learning and postlearning periods relative to the control (Fig. 2*C*; *SI Appendix*, Table S1 shows activation locations and *t*-values). At the single-subject level, we assessed the left hemispheric activations associated with speech and nonspeech vocal responses separately. We observed that individual left ventral area 44 peaks (Fig. 2*C*, dark blue circles) during both learning (speech vocal peaks, 14 of 18 subjects; nonspeech vocal peaks, 9 of 18 subjects) and postlearning (speech vocal peaks, 10 of 18 subjects; nonspeech vocal peaks, 12 of 18 subjects) were consistently located in the pars opercularis region bounded anteriorly by the anterior ascending ramus of the lateral (Sylvian) fissure, posteriorly by the inferior precentral sulcus, dorsally by the inferior frontal sulcus, and ventrally by the lateral (Sylvian) fissure (Fig. 2*C*). This finding indicated that both the speech and nonspeech vocal response selections recruited the same ventral area 44. The pars opercularis, where area 44 lies, is precisely the region that electrical stimulation of which yields speech arrest during brain surgery (18).

### Functional Dissociations in Broca’s Region (Dorsal and Ventral Area 44 and Area 45) during Cognitive Selections of Manual, Orofacial, and Vocal Actions.

Across all response conditions, we observed increased activity in the dorsal part of area 44 in the left hemisphere as subjects selected their responses during learning (Fig. 2 *A*–*C*; *SI Appendix*, Table S1 shows activation location and *t*-value), but not during the postlearning period (*SI Appendix*, Table S1). These results suggest that the dorsal area 44 contributes specifically to the learning of conditional if/then rules, and, critically, in an effector-independent manner. By contrast, ventral area 44 is effector-dependent as it is recruited during the cognitive selection of orofacial and vocal (nonspeech and speech) responses—but not of manual responses—during both the learning and postlearning periods.

In contrast to the involvement of left ventral area 44 in orofacial and both speech and nonspeech vocal conditional selections, left area 45 showed increased activity only for vocal conditional selections (pooled across nonspeech and speech vocal responses) during the learning but not the postlearning period (Fig. 2*C* and *SI Appendix*, Table S1). Single-subject level analysis revealed that left area 45 peaks (Fig. 2*C*, yellow circles) during learning (speech vocal peaks, 11 of 18 subjects; nonspeech vocal peaks, 9 of 18 subjects) are consistently located in the pars triangularis of the inferior frontal gyrus, which is bounded posteriorly by the anterior ascending ramus of the lateral (Sylvian) fissure, dorsally by the inferior frontal sulcus, and ventrally by the lateral (Sylvian) fissure (Fig. 2*C*). This is exactly where granular prefrontal area 45 lies (15, 25–27).

### The Dorsomedial Frontal Cortex Is Involved during the Learning of Conditional Visuo-Motor Associations, but Not during the Postlearning Performance of these if/then Selections.

**Fig. 3.**
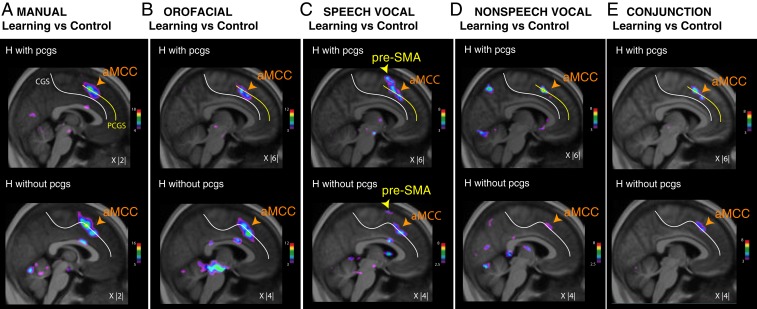
Dorsomedial frontal activations associated with the learning of manual, orofacial, and vocal conditional associations during response selection. Group-level results of increased activity during the learning period versus control trials in hemispheres (H) with a cingulate (cgs) and a paracingulate sulcus (pcgs; *Top*) and with cgs only (*Bottom*) during manual (*A*), orofacial (*B*), speech vocal (*C*), and nonspeech vocal (*D*) response selection. (*E*) Conjunction analysis between the contrasts presented in *A*–*D* for hemispheres with (*Top*) and without pcgs (*Bottom*). The color scales represent the range of the *t*-statistic values. The *X* values correspond to the mediolateral level of the section in the MNI space.

The comparison between the BOLD signal during the response selection epochs in learning versus control trials and postlearning versus control trials revealed increased activity in the dorsomedial frontal cortex (DMF) only during the learning period, not during the postlearning period (Fig. 3 and *SI Appendix*, Table S2). Thus, in contrast to the posterior lateral frontal cortex, the DMF is not involved in the postlearning selection of conditional responses from various effectors.

In view of the significant intersubject and interhemispheric sulcal variability observed in the medial frontal cortex (28, 29), we performed subgroup analyses of the learning minus control comparison (during response selection) separately for hemispheres displaying a paracingulate sulcus (pcgs) and hemispheres without pcgs (Fig. 3 and *SI Appendix*, Table S2). In both the pcgs and the no-pcgs subgroups, we observed two foci of increased activity in the anterior midcingulate cortex (aMCC) across all four response conditions (Fig. 3 *A*–*D*; *SI Appendix*, Table S2 shows peak locations and *t*-values). As demonstrated by a conjunction analysis (Fig. 3*E*), the two aMCC peaks occupy the same locations across response modalities. Importantly, our results also revealed that increased aMCC activity was consistently observed in the pcgs when this sulcus was present, and in the cgs when the pcgs was absent (Fig. 3). These results suggest that the aMCC is involved in the conditional selection of all effector types during learning when the learning is based on auditory speech or nonspeech vocal feedback.

Importantly, the pre-SMA showed increased response selection activity only during the learning of visuo-speech vocal associations (Fig. 3*C* and *SI Appendix*, Table S2), and not for learning associations between visual stimuli and the other response effectors (manual, orofacial, and nonspeech vocal). This point is confirmed in Fig. 3*E*, which shows that the pre-SMA does not display increased activity in the conjunction analysis across the various response conditions. The border between SMA and pre-SMA was defined by the coronal section at the anterior commissure (10, 11).

### The VLF–DMF Network Is Involved in Auditory Vocal Feedback Analysis during Conditional Associative Learning.

To identify the brain regions associated with the analysis of auditory nonspeech vocal and speech vocal feedback during the learning of conditional relations, we contrasted, respectively, (i) the BOLD signal during the nonspeech vocal feedback epochs in learning versus control trials with the same motor effector and (ii) the BOLD signal during the speech vocal feedback epochs in learning versus control trials with the same motor effector.

**Fig. 4.**
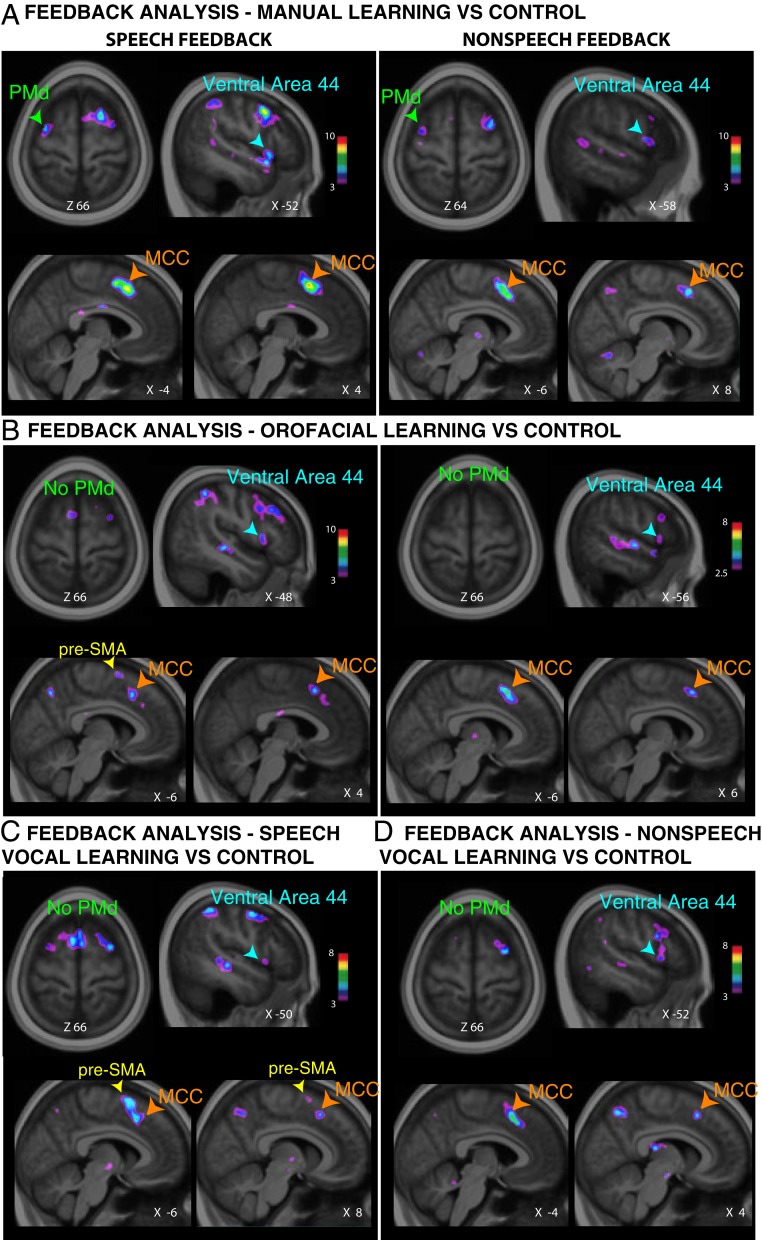
Posterior-lateral frontal and dorsomedial frontal activations associated with speech versus nonspeech vocal feedback (FB) analysis during conditional associative learning. Group analysis results displaying increased activity during the analysis of speech (*Left*) and nonspeech vocal (*Right*) feedback during the learning of (*A*) manual, (*B*) orofacial, (*C*) speech vocal, and (*D*) nonspeech vocal conditional associations. Note that the type of vocal feedback is matched to the type of vocal response for the speech and nonspeech vocal conditions. The color scales represent the ranges of the *t*-statistic values. The *X* and *Z* values correspond to the mediolateral and dorsoventral levels of the section in the MNI space, respectively.

During the learning of visuo-manual associations, the analysis of nonspeech and speech vocal feedback showed increased activity in ventral area 44 and the MCC (Fig. 4*A* and *SI Appendix*, Table S3). Additionally, we observed increased activity in the PMd during the processing of both speech and nonspeech vocal feedback (Fig. 4*A* and *SI Appendix*, Table S3). This finding was congruent with previous research (22) that demonstrated increased PMd activity as subjects processed visual behavioral feedback during visuo-manual associative learning.

During the learning of visuo-orofacial associations, the analysis of nonspeech and speech vocal feedback also showed increased activity in ventral area 44 and the MCC (Fig. 4*B* and *SI Appendix*, Table S3). In contrast to the visuo-manual condition, there was no increased activity in the PMd. Finally, there was increased activity in the pre-SMA only during the processing of speech feedback, and not nonspeech feedback (Fig. 4*B* and *SI Appendix*, Table S3).

During the learning of visuo-vocal (both speech and nonspeech) associations, the analysis of nonspeech and speech vocal feedback also showed increased activity in ventral area 44 and the MCC (Fig. 4 *C* and *D* and *SI Appendix*, Table S3). In contrast to the visuo-manual condition, there was no increased activity in the PMd. Finally, there was increased activity in the pre-SMA only during the processing of speech feedback and not nonspeech feedback (Fig. 4 *C* and *D* and *SI Appendix*, Table S3).

In summary, during conditional associative learning, a common set of regions including ventral area 44 and the MCC is involved in the analysis of both speech and nonspeech vocal feedback to drive the learning of manual, orofacial, nonspeech vocal, and speech vocal conditional relations. The PMd region appears to be specifically recruited in feedback analysis for learning associations involving manual, but not orofacial and vocal, responses. Interestingly, the pre-SMA appeared to be specifically recruited for the processing of speech vocal feedback for learning orofacial and vocal (nonspeech and speech) conditional associations. The latter finding suggests that the pre-SMA has a particular role in exerting cognitive control on vocal and orofacial responses and in performance based on speech vocal feedback specifically.

**Fig. 5.**
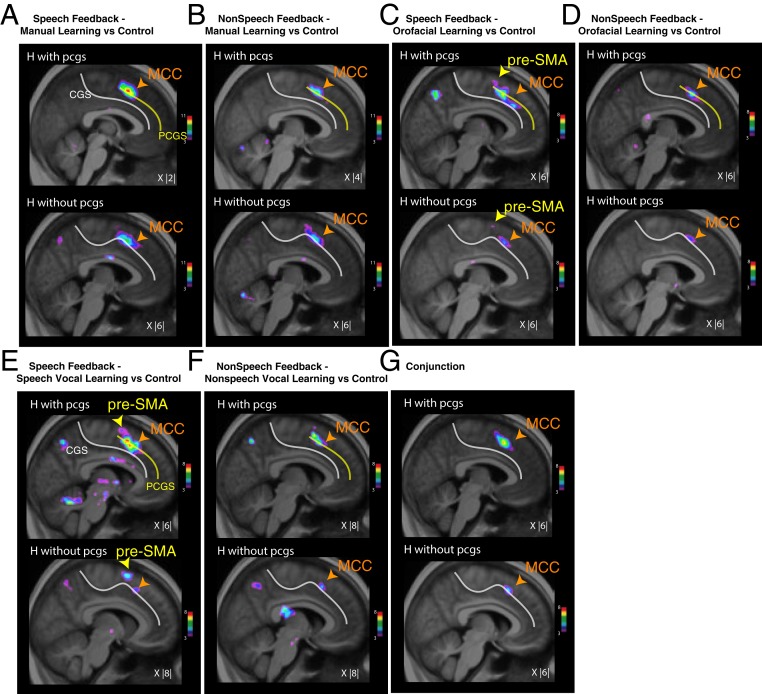
Dorsomedial frontal activations associated with auditory vocal feedback (FB) analysis during conditional associative learning in hemispheres (H) with or without a paracingulate sulcus. Group analysis results showing increased activity during auditory vocal feedback analysis in the learning period versus control trials in hemispheres with a cingulate (cgs) and a paracingulate sulcus (pcgs; *Top*) and with cgs only (*Bottom*). (*A*) Manual responses with speech feedback. (*B*) Manual responses with nonspeech feedback. (*C*) Orofacial responses with speech feedback. (*D*) Orofacial responses with nonspeech feedback. (*E*) Speech vocal responses with speech feedback. (*F*) Nonspeech vocal responses with nonspeech feedback. (*G*) Conjunction analysis between the contrasts presented in *A*–*F* for hemispheres with (*Top*) and without pcgs (*Bottom*). The color scales represent the ranges of the *t*-statistic values. The X values correspond to the mediolateral levels of the section in the MNI space, respectively.

To identify the precise location of the MCC region involved in auditory vocal processing, we compared the BOLD signal during the analysis of speech and nonspeech vocal feedback in learning versus control trials in hemispheres with and without a pcgs (*Methods*). The results indicated that speech and nonspeech vocal feedback processing is occurring in the pcgs when present and in the cgs when the pcgs is absent (Fig. 5). The same region is involved across all response-type effectors (manual, Fig. 5 *A* and *B*; orofacial, Fig. 5 *C* and *D*; speech vocal, Fig. 5*E*; nonspeech vocal, Fig. 5*F*) as shown by the conjunction analysis (Fig. 5*G*).

### The MCC Region That Is Involved in Auditory Vocal Feedback Analysis Is the Face Motor Representation of the Cingulate Motor Area.

**Fig. 6.**
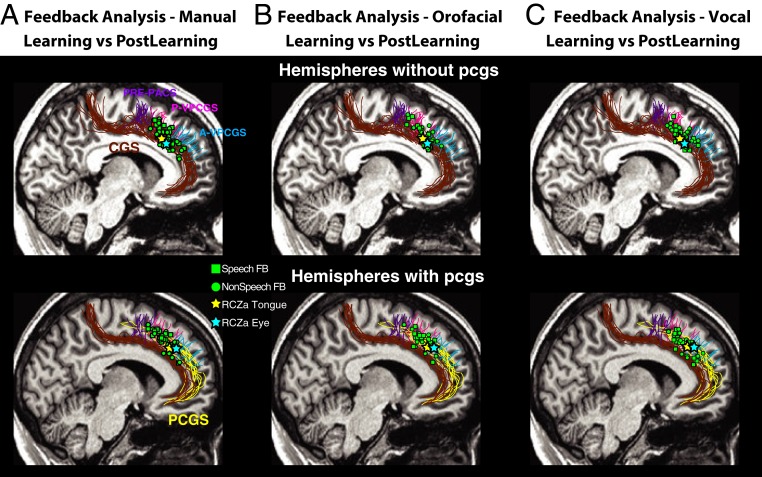
MCC activations associated with auditory vocal feedback (FB) analysis in relation to the face motor representation of the anterior rostral cingulate motor zone (RCZa). Comparison between the BOLD signal at the occurrence of speech (squares) and nonspeech (circles) feedback during conditional associative learning versus postlearning in individual subjects (each square/circle represents the location of increased activity of one subject) and in hemispheres displaying both a cingulate sulcus (cgs) and a paracingulate sulcus (pcgs; *Top*) and in hemispheres without a pcgs (*Bottom*). (*A*) Activations in the manual condition. (*B*) Activations in the orofacial condition. (*C*) Activations in the vocal condition. Green squares and circles correspond to speech and nonspeech feedback, respectively. Yellow and blue stars represent average locations of the tongue and eye motor representations in the RCZa that were derived from a motor mapping task in ref. 31. Abbreviations: cgs, cingulate sulcus; pcgs, paracingulate sulcus; prepacs, preparacentral sulcus; a/p-vpcgs, anterior/posterior vertical paracingulate sulcus.

To identify precisely the brain regions associated with the analysis of vocal feedback during learning, we compared the BOLD activity during the occurrence of feedback in learning versus the exact same feedback period in postlearning trials. This specific contrast was used to relate findings to previous studies assessing the neural basis of feedback analysis in tasks that included a learning and a postlearning period (23). We assessed the feedback-related brain activity associated with the six possible response-feedback combinations: 1) manual responses with nonspeech feedback; 2) manual responses with speech feedback; 3) vocal responses with nonspeech feedback; 4) vocal responses with speech feedback; 5) orofacial responses with nonspeech feedback; and 6) orofacial responses with speech feedback (Fig. 6). As shown in Fig. 6, activation peaks associated with the processing of speech (green squares) and nonspeech vocal feedback (green circles) were consistently located in the same MCC region across all three response modalities. Specifically, individual increased activities were found close to the intersection of the posterior vertical paracingulate sulcus (p-vpcgs) with the cgs when there was no pcgs and with the pcgs when present. This position corresponds to the known location of the face motor representation of the anterior rostral cingulate zone (RCZa; Fig. 6) (30). To confirm that these vocal feedback processing-related activation peaks corresponded to the RCZa “face” motor representation, we compared their locations with the average activation peaks corresponding to the tongue (average MNI coordinates in pcgs hemispheres, −8, 28, 37; no-pcgs hemispheres, −4, 16, 34) and eye RCZa motor representations (average MNI coordinates in pcgs hemispheres, −8, 32, 36; no-pcgs hemispheres, −7, 23, 36) obtained in a previous study from the same set of subjects (31). As displayed in Fig. 6, the feedback-related peaks obtained in the present study were located close to the average RCZa face (eye and tongue) representation.

## Discussion

One of the most intriguing observations in cognitive neuroscience is that a frontal cortical network that, in the human brain, is involved in the control of speech also exists in the brain of nonhuman primates (2, 7, 8). The present study demonstrates that, in the human brain, this network expresses a basic function that might be common to all primates and, in the language-dominant hemisphere of the human brain, is used for speech production. Specifically, as in the macaque, the basic role of area 44 in the human brain is to exert cognitive control on orofacial and nonspeech vocal responses, whereas the basic role of the midcingulate cortex is to analyze nonspeech vocal feedback driving response adaptation. By contrast, cognitive control of human-specific speech vocal information requires the additional recruitment of area 45 and pre-SMA.

### The Ventrolateral Frontal (VLF) Network.

First, within the VLF network, ventral area 44 was specifically involved in the cognitive conditional (i.e., rule-based) selection between competing orofacial acts, as well as nonspeech vocal and speech vocal acts, during both the acquisition and execution of such responses. Importantly, area 44 was not involved during the learning, selection, and execution of conditional manual responses, which, instead, recruited the dorsal premotor cortex (PMd), consistent with the previous demonstration of the role of PMd in manual response selection (22). In individual subjects, ventral area 44 activity peaks were consistently situated in the pars opercularis of the inferior frontal gyrus, i.e., the region bordered by the inferior frontal sulcus, the lateral fissure, the anterior ascending ramus of the lateral fissure, and the inferior precentral sulcus. This is exactly the region where dysgranular area 44 lies (25, 26, 32, 33). Thus, these results provide strong support to the hypothesis that area 44 plays a critical role in the cognitive rule-based selection of orofacial and vocal nonspeech and vocal speech responses (27). This hypothesis is based on the anatomical connectivity profile of area 44: strong connections with the precentral motor orofacial region and the two granular prefrontal cortical areas that are in front of and above it (area 45 and the middorsolateral area 9/46v). It also receives somatosensory inputs from the parietal operculum, insula, and the rostral inferior parietal lobule, and has strong connections with both the lateral and medial premotor regions (26, 34). This connectivity profile indicates that dysgranular area 44 is ideally placed to mediate top-down high-level cognitive decisions (i.e., from the prefrontal cortex) on vocal and orofacial motor acts that will ultimately be executed by the orofacial precentral motor region of the brain and result in speech output. Moreover, several functional neuroimaging studies have implicated area 44 in speech control and production (35), and, as we have known from classic studies, electrical stimulation of the pars opercularis of the inferior frontal gyrus where area 44 lies results in speech arrest (18). However, the present findings appear to be inconsistent with the notion that area 44 is involved in polymodal action representations (e.g., refs. 36 and 37), as we observed ventral area 44 activations associated with selections of vocal and orofacial, but not manual, actions, indicating a modality-specific involvement of ventral area 44. This is likely due to the fact that our conditional associative task involved more basic and non–object-related, visually guided action representations rather than “object-use”–related action representations. As such, our work has highlighted a potentially differential role of area 44 in object-related versus non–object-related actions. More work would be required to confirm the precise nature of the role of area 44 in object-related versus non–object-related action representations.

It is of considerable interest in terms of the evolution of the primate cerebral cortex that the anatomical homologs of the VLF cortical areas exist in NHPs (7, 8) and are implicated in aspects of cognitive vocal control (2). The present results suggest that the role of ventral area 44 in cognitive selection of orofacial and vocal acts could be conserved across primates. The observation that the ventral area 44 is involved in the analysis of nonspeech vocal and verbal feedback during conditional associative learning across all response modalities is congruent with both human and monkey studies showing that the VLF is not only involved in the production of vocal responses but also in the processing of auditory vocal information during vocal adjustments. In monkeys, Hage and Nieder (38) found that the same ventrolateral prefrontal neurons that are involved in conditioned vocal productions also responded to auditory information. They suggested that this mechanism could underlie the ability of monkeys to adjust vocalizations in response to environmental noise or calls by conspecifics. In human subjects, Chang and colleagues (39) have shown, via intracortical recordings, that, as subjects adjusted their vocal productions in response to acoustic perturbations, the ventral prefrontal cortex reflected compensatory activity changes that were correlated with both the activity associated with auditory processing and the magnitude of the vocal pitch adjustment. Functional neuroimaging investigations have also shown that human area 44 shows increased activity during both the processing of articulatory/phonological information and the production of verbal responses (35). These findings suggest that a basic role of area 44 in orofacial and vocal control in NHPs has been adapted in the language-dominant hemisphere of the human brain to serve speech output. Thus, area 44 (which lies immediately anterior to the ventral premotor cortex that controls the orofacial musculature) is shown here to be the fundamental area regulating orofacial/vocal output selections, regardless of whether these selections involve just orofacial movements, nonspeech vocal, or speech vocal responses and regardless of whether these selections occur during the learning or postlearning periods. The present findings suggest that dysgranular area 44 may be the critical regulator of vocal output and, therefore, provide an explanation why interference with its function (as in electrical stimulation during brain operations) results in the purest form of speech arrest (18). By contrast, electrical stimulation of the ventral precentral motor region directly controlling the orofacial musculature leads both to “vocalizations,” i.e., involuntary and meaningless vocal output, as well as interference with normal speech (18).

Importantly, the present study demonstrated a major difference between the activations of areas 44 and 45, although both these cytoarchitectonic areas are considered to be part of Broca’s region in the language-dominant hemisphere (33). There are, however, major differences in the cytoarchitecture and connectivity of these areas (8, 32–34). Unlike dysgranular area 44, which lies immediately anterior to the orofacial representation of the motor precentral gyrus, and was shown here to be involved in rule-based orofacial, nonspeech vocal, and speech vocal response selections both during the learning and the postlearning periods, activation in granular prefrontal area 45 was related only to the conditional selection of vocal nonspeech and speech responses, and only during the learning period, namely the period when the conditional relations are not well learned and the subject must, therefore, engage in active mnemonic retrieval. This finding is consistent with the hypothesis that area 45 is critical for active selective controlled memory retrieval (26, 27). There is functional neuroimaging evidence of the involvement of area 45 in the controlled effortful mnemonic retrieval of verbal information, such as the free recall of words that have appeared within particular contexts (14). A more recent study has shown that patients with lesions to the ventrolateral prefrontal region, but not those with lesions involving the dorsolateral prefrontal region, show impairments in the active controlled retrieval of the contexts within which words were presented (40).

The present findings regarding the differential involvement of the two cytoarchitectonic areas that comprise Broca’s region are consistent with the hypothesis that the prefrontal granular component, i.e., area 45, in the left hemisphere, is the critical element for the selective controlled retrieval of verbal information, which is then turned into speech utterances by the adjacent dysgranular area 44, leading to the final motor output via the precentral orofacial region (27). With its specific role in selective cognitive retrieval, area 45 in the language-dominant hemisphere of the human brain came to support speech production by the retrieval of high-level multisensory semantic information that will be turned into speech utterance selections by transitional dysgranular area 44 and into final motor output (i.e., control of the effectors) by the ventral precentral orofacial motor region. Indeed, a recent functional neuroimaging study in the human brain combined with diffusion tensor imaging-based tractography has presented evidence that the temporofrontal extreme capsule fasciculus that links area 45 with the anterior temporal lobe is the critical pathway of a ventral language system mediating higher-level language comprehension (41). It is of considerable interest that the temporofrontal extreme capsule fasciculus was first discovered in the macaque monkey (42, 43). This pathway, which must not be confused with the uncinate fasciculus, links area 45 and other high-level prefrontal areas with the lateral anterior and middle temporal region that integrates multisensory information. This critical high-level frontotemporal interaction is most likely the precursor of a system for the controlled selective retrieval of specific auditory, visual, multisensory, and context-relevant information that, in the human brain, came to mediate semantic information and exchange between the anterior to middle temporal lobe and the ventrolateral prefrontal cortex via the extreme capsule (41).

In monkeys, the ventrolateral prefrontal cortex (which includes areas 45 and 47/12) has been found to contain neurons that respond to both species-specific vocalizations and faces (38, 44), consistent with the suggested role of this prefrontal region in the active cognitive selective retrieval and integration of audiovisual information. Indeed, the anteroventral area 45 and the adjacent area 47/12 of the ventrolateral frontal cortex do link with the visual processing area TE in the midsection of the inferior temporal region (8). Notably, the macaque TE region is involved in the processing and recognition of novel shapes (45) and is often regarded as a putative homolog to the visual word form area (VWFA; ref. 46) that is found in the human ventral temporal cortex and also anatomically linked with area 45 (47). As such, from monkeys to humans, the granular areas 45 and 47/12 of the ventrolateral frontal cortex might have evolved from the retrieval and integration in the anterior temporal region of basic audiovisual communicative information (e.g., shapes, faces, vocalizations, including multisensory information) to more complex multimodal inputs that are inherent in speech and semantic processing.

The present results also demonstrated a dorsoventral functional dissociation within the pars opercularis (area 44) itself. Distinct from the ventral area 44 discussed earlier, dorsal area 44 was involved specifically during the learning period of visuo-motor conditional associations across all response modalities, but not during the execution of learned associations. In support of this dissociation, recent parcellations of the pars opercularis on the basis of cytoarchitecture and receptor architecture (25, 32), as well as connectivity (48), have suggested that area 44 can be further subdivided into dorsal and ventral parts. Recent neuroimaging studies have also shown functional dissociations between the dorsal and ventral area 44, although the precise roles attributed to the two subregions are currently still debated. For instance, Molnar-Szakacs and colleagues (49) found that increased activity in the dorsal area 44 (and also area 45; see ref. 50) was related to both action observation and imitation, while activation in ventral area 44 was related only to action imitation. In agreement, Binkofski and colleagues (51) showed that the ventral, but not the dorsal, area 44 was implicated during movement imagery. Finally, during language production, the ventral area 44 was shown to be involved in syntactic processing (52) and comprehension (53), while the dorsal area 44 was involved in phonological processing (54). Thus, our findings are clearly in agreement with the emerging view that the pars opercularis can be subdivided into dorsal and ventral parts.

### The Dorsomedial Frontal (DMF) Network.

The VLF and DMF networks are interconnected (2, 8). What might be the specific contributions of the areas that comprise the DMF network? Our findings demonstrate that the MCC is involved during the learning of conditional responses based on auditory nonspeech and speech vocal feedback. Note that the MCC was not involved in response selection during the postlearning period, indicating its specific role in adaptive learning, in agreement with previous studies (22, 23, 55). Furthermore, the role of the MCC during learning of visuo-motor conditional associations is not effector-specific: the same MCC region is activated during conditional associative learning regardless of whether the responses are manual, orofacial, nonspeech vocal, or speech vocal. Importantly, subject-by-subject analyses further indicated that the activation focus in the MCC, for both nonspeech and speech vocal feedback, corresponds to the “face” motor representation within the anterior MCC (RCZa). As such, our findings indicate that the “face” motor representation of RCZa, within the MCC, contributes to the processing of auditory vocal and verbal feedback for behavioral action adaptation. Consistent with our results, accumulating evidence from both monkey and human functional investigations converges on the role of the primate MCC in driving behavioral adaptations via the evaluation of action outcomes (21, 55, 56). Importantly, based on a review of the locations of outcome-related and motor-related activity in the monkey and human MCC, Procyk and colleagues (21) reported an overlap between the locations for the evaluation of juice-rewarded behavioral outcomes and the face motor representation in the monkey rostralmost cingulate motor area (CMAr), strongly suggesting that behavioral feedback evaluation in the MCC is embodied in the CMAr motor representation corresponding to the modality of the feedback. The present study supports this hypothesis, showing that adaptive auditory feedback is being processed by the face motor representation in the human homolog of the monkey CMAr, i.e., RCZa. Furthermore, a recent fMRI study in the macaque monkey has shown that the face representation in the DMF system is involved in the perception of the communicative intent of another primate (57). Thus, the VLF system is involved in the high-level specific and context-relevant information retrieval (prefrontal areas 45 and 47/12), cognitive rule-based conditional selections of orofacial and vocal actions (dysgranular area 44), and final execution of these acts via the precentral orofacial/vocal motor zone (areas 6 and 4). By contrast, the DMF system is involved in the process of learning the rules based on adaptive nonspeech and speech vocal feedback processed in the orofacial face representation of the DMF system that also includes facial communicative intent.

### The Special Role of the Pre-SMA.

The present study demonstrated that the pre-SMA is selectively recruited during the learning of conditional speech (but not nonspeech vocal, orofacial, or manual) response selections based on verbal (but not nonspeech vocal) feedback. These findings highlight the special role of pre-SMA in the learning of verbal responses and the processing of verbal feedback for such learning. The current literature suggests that the pre-SMA is involved in the temporal sequencing of complex motor actions (58, 59) and the learning of associations between visual stimuli and these action sequences (60, 61). A possible explanation of the pre-SMA’s unique involvement in the learning of visuo-verbal associations in the present study might be that the verbal responses involve the sequencing of more complex motor acts (i.e., involving multiple sounds), whereas manual (single button presses), orofacial (single mouth movements), and vocal responses (single vowel sounds) involve less complex and individual motor actions. In support of the pre-SMA’s role in verbal processing, Lima and colleagues (62) have shown that the pre-SMA is often engaged in the auditory processing of speech. Importantly, these investigators also suggested that the pre-SMA is involved in the volitional activation/retrieval of the specific speech motor representations associated with the perceived speech sounds. This could explain our observation that the pre-SMA was active during both the processing and selection of verbal responses during learning. The role of the pre-SMA in the learning of context–motor sequence associations that is observed in the macaque (63) appears to be conserved in the human brain. Although NHPs do not produce speech, it has been shown that the pre-SMA in monkeys is associated with volitional vocal production (2): stimulation in the pre-SMA produces orofacial movements (64). Lesions of the pre-SMA region lead to increased latencies of spontaneous and conditioned call productions (65). Based on these findings, it appears that the role of pre-SMA in the volitional control of orofacial/vocal patterns may have been adapted in the human brain for the control of speech patterns via context–motor sequence associations.

### How Might the VLF–DMF Network Have Evolved to Support Human Speech?

Together, the results of the present investigation demonstrate that, within the human VLF–DMF network, ventral area 44 and MCC appear to subserve basic functions in primate cognitive vocal control: ventral area 44 is involved in the cognitive rule-based selection of vocal and orofacial actions, as well as in the active processing of auditory-vocal information; by contrast, the MCC is involved in the evaluation of vocal/verbal feedback and communicative intent that leads to behavioral adaptation in learning conditional associations between vocal/orofacial actions and arbitrary external visual stimuli. Indeed, in a previous review (2), we have argued that the aforementioned functional contributions of area 44 and MCC are generic across primates based on anatomical and functional homologies of these regions in cognitive vocal control. Within the human VLF–DMF network, area 45 and the pre-SMA may be regions that, in the language dominant hemisphere, have specialized for verbal processing: area 45 is recruited for the selective controlled retrieval of verbal/semantic information that will be turned into orofacial action by area 44, while the pre-SMA is specifically involved in driving verbal action selections based on auditory verbal feedback processing.

Another important adaptation that could have contributed to the emergence of human speech capacities is the emergence of a cortical laryngeal representation in the human primary motor orofacial region that afforded increased access to fine-motor control over orolaryngeal movements (66). As such, ventral area 44, with strong connections to the primary motor orofacial region via the ventral premotor cortex, would be in a position to exercise control via conditional sensory–vocal associations over a wider range of orolaryngeal actions. The pre-SMA, which is strongly linked to the primary motor face representation, via the SMA, would also be able to build context–motor sequence associations with complex speech motor patterns and activate them based on their auditory representations. The MCC, which is directly connected to the ventral premotor area, would be able to influence orolaryngeal adaptations, based on feedback evaluation, at the fine motor level. Finally, area 45 would provide semantic and other high-level information selectively retrieved from lateral temporal cortex and posterior parietal cortex that would bring the VLF–DMF network in the service of higher cognition in the language-dominant hemisphere of the human brain (27, 33). These adaptations could explain the expanded capacity of the human brain to generate flexibly and modify vocal patterns.

## Methods

### Subjects.

A total of 22 healthy right-handed native French speakers were recruited to participate in a training session and three fMRI sessions. Data from two subjects (S2, S13) were omitted from the analyses because they had shown poor performance across the three functional neuroimaging sessions. Two other subjects (S6, S9) did not participate in any of the scanning sessions because of claustrophobia. Consequently, the final dataset consisted of 18 subjects (10 males; mean age, 26.22 y; SD, 3.12). The study was carried out in accordance with the recommendations of the Code de la Santé Publique and was approved by Agence Nationale de Sécurité des Médicaments et des Produits de Santé (ANSM) and Comité de Protection des Personnes (CPP) Sud-Est III (No EudraCT: 2014-A01125-42). It also received a *ClinicalTrials*.*gov* ID (NCT03124173). All subjects provided written informed consent in accordance with the Declaration of Helsinki.

### Experimental Paradigm.

In the present study, subjects performed three versions of the visuo-motor conditional learning and control tasks in the scanner that corresponded to three different response effectors: manual, orofacial, and vocal (nonspeech or speech; Fig. 1, *SI Appendix*, *Supplementary Methods*, and Movies S1–S3). In the visuo-manual condition, the subjects acquired associations between three finger presses on an MRI-compatible button box (Current Designs) and visual stimuli in the conditional learning task and performed instructed button hand presses in the control task. In the visuo-orofacial condition, the subjects performed the conditional learning task and control task using three different orofacial movements (Fig. 1 *B*, *Middle*). In the visuo-vocal condition (Fig. 1*B*, rightmost panel), the responses were either three different meaningless nonspeech vocal responses (“AAH,” “OOH,” “EEH”) or speech vocal responses (the French words “BAC,” “COL,” “VIS”) during the learning and control tasks and the feedback provided was either nonspeech or speech vocal, respectively. These nonspeech and speech vocal responses were selected to match, as closely as possible, the orofacial movements performed in the visuo-orofacial condition: the first orofacial action (Fig. 1*B*, top image in the orofacial panel) is almost identical to the mouth movements engaged in producing the nonspeech vocal action “AAH” and similar to the speech vocal action “BAC.” In the same manner, the second and third orofacial actions corresponded to nonspeech vocal actions “EEH” and “OHH” and speech vocal actions “VIS” and “COL,” respectively. Subjects were informed of which set of responses to use via the text color of the instructions (red, speech vocal; yellow, nonspeech vocal). To ensure optimal performance during the actual fMRI sessions, all subjects were familiarized with all three versions of the learning and control tasks in a separate training session held outside the scanner. During the training session, the subjects practiced the visuo-manual, visuo-orofacial, and visuo-vocal conditional learning tasks until they consistently met the following criteria in each version: (i) not more than one suboptimal search (i.e., trying the same incorrect response to a particular stimulus or trying a response that had already been correctly associated to another stimulus) during the learning phase and (ii) not more than one error in the postlearning phase.

#### MRI analyses.

For each subject, fMRI data from the three fMRI sessions (manual, orofacial, and vocal [speech and nonspeech]) were modeled separately. At the first level, each trial was modeled with impulse regressors at the two main events of interest: (i) response selection (RS), the 2-s epoch after the stimulus onset, during which the subject had to perform a response after stimulus presentation; and (ii) auditory feedback (FB), the 1-s epoch after the onset of auditory feedback. RS and FB epochs were categorized into either learning (RS_L_, FB_L_), postlearning (RS_PL_, FB_PL_), or control (RS_C_, FB_C_) trial events. These regressors were then convolved with the canonical hemodynamic response function and entered into a general linear model of each subject’s fMRI data. The six scan-to-scan motion parameters produced during realignment and the ART-detected motion outliers were included as additional regressors in the general linear model to account for residual effects of subject movement.

To assess the brain regions involved in the visuo-motor conditional response selection, we contrasted the blood oxygenation-level dependent (BOLD) signal during RS_L_ and RS_PL_ events, when subjects actively selected their responses on the basis of the presented stimulus, with RS_C_ events, when subjects performed instructed responses. The two main contrasts (i.e., RS_L_ vs. RS_C_ and RS_PL_ vs. RS_C_) were examined for each response version at the group level and at the subject-by-subject level. At the group level, speech and nonspeech vocal response selection trials are pooled in order to increase statistical power. To examine possible differences between nonspeech and speech vocal responses, we distinguished between the two conditions in our subject-level analyses.

To determine the brain regions involved in the processing of auditory feedback during the learning of visuo-manual, visuo-orofacial, and visuo-vocal conditional associations, we examined the contrasts between FB_L_ and FB_PL_ events and between RS_L_ and RS_PL_ in each response version to determine if distinct areas are involved in the processing of auditory feedback during the different response modalities. To determine whether speech and nonspeech vocal feedback processing recruited different brain regions, we performed the aforementioned analyses separately for each response type (manual, nonspeech and speech vocal, orofacial) with speech or nonspeech vocal feedback.

**Fig. 7.**
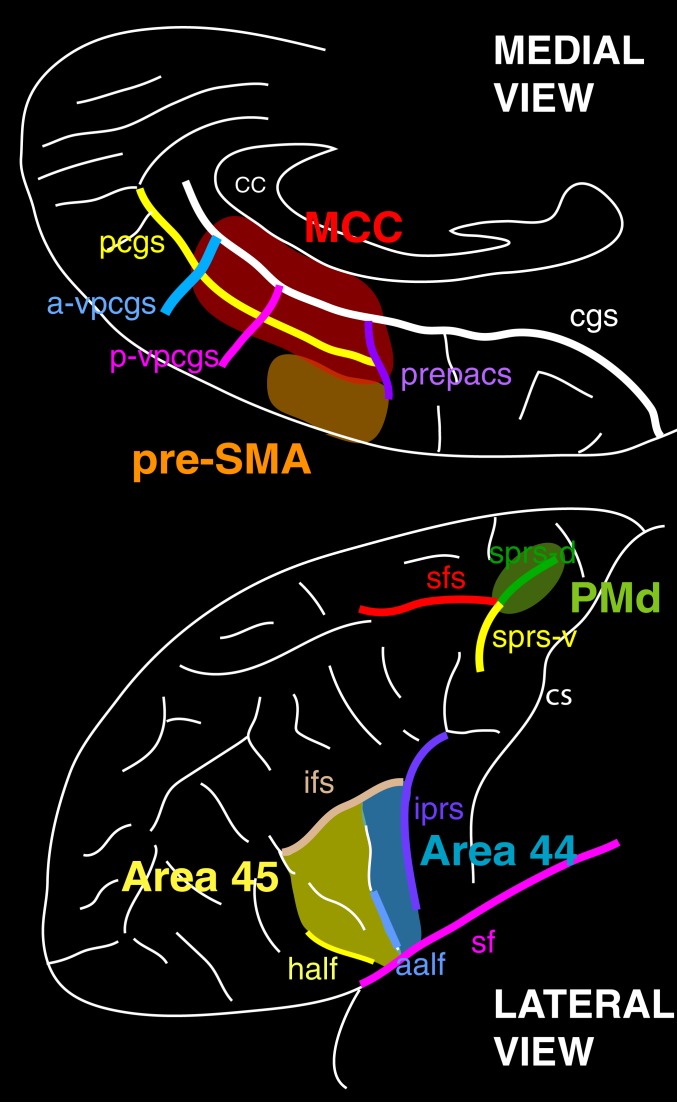
Characteristic sulci of the MCC and the pre-SMA (medial view), areas 44 and 45 in Broca’s region, and the dorsal premotor cortex (PMd; lateral view). In the PMd, the characteristic sulci are the dorsal branch of the superior precentral sulcus (sps-d), the ventral branch of the superior precentral sulcus (sps-v), and the superior frontal sulcus (sfs). Area 44 is bounded by the inferior precentral sulcus (ips), the anterior ascending ramus of the lateral (Sylvian) fissure (aalf), and the inferior frontal sulcus (ifs). Area 45 is bounded by the anterior ascending ramus of the lateral (Sylvian) fissure (aalf), the horizontal ascending ramus of the lateral (Sylvian) fissure (half), and the inferior frontal sulcus (ifs). In the medial frontal cortex, the characteristic sulci are the cingulate sulcus (cgs), the paracingulate sulcus (pcgs), and the vertical sulci joining the cgs and/or pcgs, i.e., the preparacentral sulcus (prepacs), the posterior vertical paracingulate sulcus (p-vpcgs), and the anterior vertical paracingulate sulcus (a-vpcgs). Abbreviations: CC, corpus callosum; sf, Sylvian (lateral) fissure.

Because of individual variations in cortical sulcal morphology in the dorsal premotor region (PMd), the ventrolateral Broca’s region, and the medial frontal region, these analyses were also assessed at the subject-by-subject level. In PMd, we identified activation peaks in relation to the dorsal branch of the superior precentral sulcus, the ventral branch of the superior precentral sulcus, and the superior frontal sulcus (Fig. 7). We identified activation peaks in relation to the limiting sulci of the pars opercularis where area 44 lies, i.e., the inferior precentral sulcus (iprs), the anterior ascending ramus of the lateral fissure (aalf), the horizontal ascending ramus of the lateral fissure (half), and the inferior frontal sulcus (ifs). In the medial frontal cortex, we identified activation peaks in relation to the cingulate sulcus (cgs), paracingulate sulcus (pcgs), and the vertical sulci joining the cgs and/or pcgs (i.e., the preparacentral sulcus [prepacs], and the posterior vertical paracingulate sulcus [p-vpcgs]). It should be noted that the pcgs is present in 70% of subjects at least in one hemisphere, and several studies have shown that the functional organization in the cingulate cortex depends on the sulcal pattern morphology. We therefore also performed subgroup analyses of fMRI data in which we separated hemispheres with a pcgs from hemispheres without a pcgs (see Amiez et al. [23] for the full description of the method).

For the group, subgroup, and individual subject analyses, the resulting *t* statistic images were thresholded using the minimum given by a Bonferroni correction and random field theory to account for multiple comparisons. Statistical significance for the group analyses was assessed based on peak thresholds in exploratory and directed search and the spatial extent of consecutive voxels. For a single voxel in a directed search, involving all peaks within an estimated gray matter of 600 cm^3^ covered by the slices, the threshold for significance (*P* < 0.05) was set at *t* = 5.18. For a single voxel in an exploratory search, involving all peaks within an estimated gray matter of 600 cm^3^ covered by the slices, the threshold for reporting a peak as significant (*P* < 0.05) was *t* = 6.77 (67). A predicted cluster of voxels with a volume extent >118.72 mm^3^ with a *t*-value > 3 was significant (*P* < 0.05), corrected for multiple comparisons (67). Statistical significance for individual subject analyses was assessed based on the spatial extent of consecutive voxels. A cluster volume extent >444 mm^3^ associated with a *t*-value >2 was significant (*P* < 0.05), corrected for multiple comparisons (67).

### Data and Code Availability.

The raw neuroimaging and behavioral data used in the present analyses are accessible online: https://zenodo.org/record/3583091 (68). Experimental codes are available upon request from the corresponding authors.

## Supplementary Material

Supplementary File

Supplementary File

Supplementary File

Supplementary File
