# Supplementary material for: Cognitive control of orofacial motor and vocal responses in the ventrolateral and dorsomedial human frontal cortex
Source: Proc Natl Acad Sci U S A. 2020 Feb 14;117(9):4994–5005. doi: 10.1073/pnas.1916459117 (PMC7060705; doi:10.1073/pnas.1916459117)
Supplement: Supplementary File [file pnas.1916459117.sapp.pdf]

Supplementary Appendix for:

Cognitive control of orofacial motor and vocal responses in the ventrolateral and dorsomedial human frontal cortex

Kep Kee Loh, Emmanuel Procyk, Rémi Neveu, Franck Lamberton, William D. Hopkins, Michael Petrides, and Céline Amiez

Corresponding Authors :
Céline Amiez: celine.amiez@inserm.fr
Kep Kee Loh: kep-kee.loh@univ-amu.fr

This PDF file includes:

Supplementary Methods
Supplementary Results
Figure S1
Tables S1 to S4
Legends for Movies S1 to S3
SI References

Other supplementary materials for this manuscript include the following:

Movies S1 to S3

Supplementary Methods

Behavioral data acquisition. In the visuo-manual fMRI session, subjects responded directly in the task within the scanner via an MRI-compatible button box (Current Designs, Philadelphia, USA) connected to the Presentation computer. Raw trial data, including trial events, reaction times and percent correct responses were extracted directly from the Presentation logfiles.

In the visuo-vocal fMRI session, an MRI-compatible microphone (MO 2000 model, Seinnheiser Electronic, Germany) was installed on the head-coil, near the subjects' mouths, to record their vocal responses during the task. Outside the scanner, an experimenter monitored the subjects' vocal responses in real-time and responded to the task via keyboard presses on the Presentation computer. A Biopac MP150 system (Biopac Systems Inc, Goleta, CA) was used to acquire simultaneously, and synchronize: 1) the analog audio signals from the microphone, 2) task event signals from Presentation, and 3) TTL signals from the MRI scanner. After the experiment, the subjects' actual vocal response onsets were computed from the analog audio signal (time-synchronized to task events and MRI pulses) using a customized onset detection algorithm (written by R. Neveu and available upon request) implemented in Matlab (www.mathworks.com). The detected vocal responses in the audio recordings were subsequently used to verify the responses made by the experimenter during the task (logged by the Presentation software). The trial event timings were acquired directly from the Presentation logfiles.

In the visuo-orofacial fMRI session, a home-made video-camera was used to record the subjects' mouth responses as they performed the task in the scanner. The camera was installed outside the scanner tunnel and positioned to provide a view of the subjects' mouth from a mirror fixed on the head coil. As in the visuo-vocal experiment, an experimenter responded to the task outside the scanner according to the subjects' mouth responses. The Biopac system was also used to acquire the video data, in parallel with task event and MRI TTL signals. A customised Matlab program (written by K. Loh and available upon request) was used to synchronize the video recordings with the timings of the task events, and subsequently, allow the experimenter to playback manually each video segment frame-by-frame to determine the onset of each orofacial response. The orofacial responses detected from the video were cross-checked with the responses made by the experimenter during the task. The trial event onsets were acquired directly from the Presentation logfiles.

MRI data acquisition and preprocessing. Scanning was performed on a 3T Siemens Magnetom Prisma MRI Scanner (Siemens Healthcare, Erlangen, Germany). To minimize movements during the motor tasks, the subjects' heads were tightly cushioned throughout the acquisition. The main experimental protocol consisted of three MRI sessions (~2hrs), each involving a different version of the visuo-motor conditional learning and control tasks (visuo-manual, visuo-orofacial, and visuo-vocal). For each fMRI session, subjects performed four to six experimental runs in the scanner. Experimental runs were programmed and presented using Presentation (Neurobehavioral systems). Visual stimuli were presented via an LCD projector with a mirror system. Auditory feedback was delivered via MRI-compatible earphones (Siemens Healthcare, Erlangen, Germany).

The start of each run was synchronized to the 5th TTL pulse from the MRI scanner following scan initiation. Each run began with a fixation task followed by two motor mapping task blocks involving either: hand and eye movements (visuo-manual session), speech and nonspeech vocal responses (visuo-vocal session) or mouth and tongue movements (visuo-orofacial session). The details of the fixation and motor mapping tasks are described in Loh et al (1). After the three fixation/motor mapping task blocks (total duration = 28.5s), subjects were presented with the six learning task blocks (3 x 2 feedback types) and six control task blocks (3 motor responses x 2 feedback types) that were randomly interleaved in each run. The total length of each run was limited to a maximum of 14.6min, yielding 400 T2*-weighted gradient echo planar EPI volumes (40 descending oblique slices, voxel resolution = 2.7mm x 2.7mm x 2.7mm, TR = 2.2s, TE = 30.0s, flip angle = 90°). The initial five volumes of each acquisition were discarded to avoid confounds of unsteady magnetization. High-resolution T1 structural images (MPRAGE, 0.9mm^3 isotropic voxels, 192 slices, TR=3.5s, TE=2.67s), together with Diffusion Tensor Imaging (DTI) and resting state functional scans (not analyzed in the current study) were each acquired in one of the three MRI sessions at the end of all experiment task runs.

Preprocessing of the MRI data was performed with Statistical Parametric Mapping software (SPM12; Wellcome Department of Cognitive Neurology, University of College London, London, UK; http://www.fil.ion.ucl.ac.uk/spm) and Matlab 15b (www.mathworks.com). First, structural and functional images were reoriented by setting their origins to the anterior commissure. The first 5 volumes of each run were excluded to remove T1 equilibrium effects. Next, within each session, we realigned all successive images to the first image of the session. Using the Artefact Detection Toolbox (ART; http://www.nitrc.org/projects/artifact_detect/), motion outliers were computed from the realigned images and realignment parameters. The realignment parameters and detected motion outliers were saved as covariates to model potential nonlinear head motion artefacts in subsequent statistical analyses. Slice-timing correction was applied with the time centre of the volume as reference. The subject-mean functional images were co-registered with the corresponding structural images using mutual information optimization. Functional and structural images were then spatially normalized into standard MNI space. Finally, functional images were smoothed using a 6-mm full-width half-maximum Gaussian kernel (2–4).

Experimental tasks. Two main experimental tasks were implemented in the current study: 1) A visuo-motor conditional learning task (Fig. 1A; Movie S3), and 2) a visuo-motor control task (Fig. 1B; Movie S1 and S2). The sequence of events was comparable for the two tasks: an instruction screen lasting for 2s informed the participant of the type of task to be performed: “Find the correct associations” (which indicated the selection of the response linked to the particular visual cue presented on each trial) during the conditional learning task or “Select the X response” (which indicated the particular response to be performed on each trial) for the control task block, respectively. Following the instructions, a black screen with a central fixation cross was presented during a jittered inter-trial interval of 0.5-8s (mean=3.5s). One of three possible visual stimuli (abstract grayscale images) was then presented on the center of the screen for 2 seconds during which the subject had to select and perform one of three possible motor responses (learning task) or perform an instructed motor response (control task).

Stimulus presentation was ordered as randomly permuted blocks of the three possible images (i.e. abc, bca, acb, etc.). After a jittered delay (0.5-6s, mean=2s), a nonspeech or speech vocal feedback (1s) was provided to inform the subject whether the response selected and performed was the correct one for the presented image (during the learning task) or the correct instructed response (during the control task). After a jittered inter-trial interval (0.5-8s, mean=3.5s), another trial started with the presentation of one of the 3 possible stimuli. In half of the task blocks, speech vocal feedback was provided (positive: "Correct"; negative: "Error"). For the remaining blocks, nonspeech vocal feedback was provided (positive: "Aha"; negative: "Boo"). The subject was informed about the type of feedback that would be provided after the performance of each response by the text color on the instruction screen at the start of each task (Speech Vocal: Yellow, Nonspeech Vocal: Red). The four different types of vocal feedback were recorded by the same male voice and processed using Audacity software.

In each visuo-motor conditional learning task block (Fig. 1A; Movie S3), the subjects had to acquire three visuo-motor conditional associations (if visual stimulus A, then motor response X, if visual stimulus B, then motor response Y, and if visual stimulus C, then motor response Z) via trial-and-error. On every trial, one of the three possible stimuli was presented and the subject had to select one of the three possible responses to perform (Response Selection). Auditory vocal feedback was then provided to inform the subject whether the selected response was the correct one for the presented stimulus. This trial-and-error learning period continued until the subject selected and performed the correct response for each stimulus. When a correct response had been performed once for each of the three visual stimuli, the task proceeded to the "post-learning" period during which the subject had to repeat, in response to the appropriate cues, each of the learnt conditional associations twice (6 trials). Note that in each visuo-motor conditional learning task block, a novel set of 3 stimuli was presented and, therefore, the subject had to learn the new stimulus-response relations.

In each visuo-motor control task block (Fig. 1C; Movies S1 and S2), the subjects were informed of the specific response to perform on the instruction screen. Subsequently, they had to perform that particular instructed response to all of the three different visual stimuli over five trials. Thus, in contrast to the learning task, during the control task the subjects did not have to select an appropriate motor response to perform based on learning of the correct stimulus-to-response arbitrary relations nor to adjust their selections based on the provided feedback. Different sets of abstract visual images were used in all learning and control task blocks. Note that in each control task block, a novel set of 3 stimuli was used.

Supplementary Results

Behavioral task performance. Three main performance measures were used from the learning and control tasks: 1) the proportion of correctly performed blocks, 2) the number of trials constituting the learning period (learning period length), and 3) the response time (RT) for each trial. First, with the proportion of correct blocks, we examined the effects of block type (learning/control), response type (manual/orofacial/non-speech vocal/speech vocal) and feedback type (speech/non-speech), and their possible interactions. A generalized linear mixed effect regression with binomial link function was performed via the *glmer* function from the *lme* package (https://cran.r-project.org/web/packages/lme4/) in R statistical software. The statistical significance of the effects was assessed with Type II Wald tests implemented via the *Anova* function from the *car* package (https://cran.r-project.org/web/packages/car). Second, for learning period length, we examined the effect of response type via a generalized linear mixed effect regression with a gaussian link function. Finally, for the trial RT data, we examined the effects of trial type (learning/post-learning/control), response type (manual/vocal/orofacial), and feedback type (speech/non-speech vocal), and their possible interactions. Only behavioral data from the correctly performed learning and control task blocks were analyzed. A generalized linear mixed effect regression with an inverse-gaussian link function was performed via the *glmer* function. The statistical significance of the effects was assessed with Type II Wald tests implemented via the *Anova* function from the *car* package.

Our generalized linear mixed-model regression analyses revealed no significant effects of response type (χ^2=3.22, *df*=3, *p*=0.36; Fig S1A) and feedback type (χ^2=0.05, *df*=1, *p*=0.83, Fig S1A) on completion rates. This finding indicated that subjects performed equally well for both the control and learning tasks when different responses were involved and when different feedbacks were provided. As expected, we observed a significant effect of task type (χ^2=82.2, *df*=1, *p*<2x10^{-16}, Fig S1A): the completion rates were higher in control versus learning blocks. As a further indication that response type had no influence on conditional-associative learning performance, we found that the mean learning period (number of trials taken to acquire the conditional associations in correctly performed blocks) did not differ across response types (χ^2=3.05, *df*=2, *p*=0.218, Fig S1B).

In terms of response selection RTs, regression analyses revealed no significant effect of the type of feedback provided (χ^2=2.43, *df*=1, *p*=0.12, Fig S1C). There was a significant main effect of response type (χ^2=2.36x10^3, *df*=3, *p*<2x10^{-16}): subjects were fastest with manual responses, followed by orofacial, and non-speech/speech vocal responses, reflecting the increasing motor complexity from finger presses to mouth movements and vocal productions. Note that RTs in the visuo-nonspeech and visuo-speech vocal conditional learning association tasks were not significantly different (*p=1.00*). As expected, there was a main effect of trial type on RTs (χ^2=1.18x10^3, *df*=2, *p*<2x10^{-16}): RTs were faster in control than learning trials, and in learning than post-learning trials. This result reflected the fact that response selection was qualitatively different between the three trial types. Finally, there was a significant interaction (χ^2=551, *df*=6, *p*<2x10^{-16}) between the response modality and trial type on the RTs. Post-hoc analyses (pairwise comparisons using *lsmeans* package with Bonferroni correction for multiple comparisons) revealed that for all modalities, control trial RTs were consistently faster than

post-learning and learning RTs ($p<0.0001$). This finding was expected since, in control trials, no cognitive selection was involved as opposed to the learning and post-learning trials. Differentiating between the various modalities, learning RTs were significantly faster than post-learning RTs in orofacial ($p<0.0001$) and manual ($p=0.016$) response conditions, but learning and post-learning RTs did not differ for nonspeech ($p=0.570$) and speech ($p=0.872$) vocal responses. This result indicated that differential cognitive mechanisms could be involved during the learning and performance of manual and orofacial conditional associations versus vocal (speech and non-speech) conditional associations.

The precentral gyrus of the insula is involved in the cognitive selection of manual, orofacial and vocal responses. An investigation by Dronkers (5) had proposed that the left precentral gyrus of the insula (PCGi) has a critical and specific role in articulatory planning. In addition, there is anatomical evidence that area 44 is connected with the insular cortex (6). Interestingly, we observed that, compared to the appropriate control, the precentral gyrus of the insula (PCGi) appears to be more active during the post-learning response selection across manual (L. PCGi: -40, 9, -6; t=8.13; R. PCGi: 40, 12, -4; t=10.37), orofacial (L. PCGi: -40, 6, -4, t=7.11; R. PCGi: 40, 6, -2; t=6.69) and vocal responses (L. PCGi: -42, 12, -4, t=4.63), indicating that this region might have a more general role in motor planning beyond articulatory actions. More recent work by Fedorenko and colleagues (7) supports this notion by showing that non-articulatory orofacial movements also result in strong PCGi activations. Here we demonstrated that the PCGi appears to be also active during manual response selection, and that, whereas the PCGi was bilaterally activated for manual and orofacial responses, only the left PCGi was activated for vocal response selection.

Lateralization in the human VLF-DMF network during cognitive orofacial and vocal control. In the present study, the involvement of the VLF and DMF network regions in the cognitive control of orofacial and vocal responses appeared to be largely, but not entirely, left-lateralized: The cognitive selection of orofacial and vocal responses (during learning and post-learning) recruits ventral area 44 only in the left hemisphere (See Table S1). Furthermore, the recruitment of the dorsal area 44, area 45 and pre-SMA during the learning of orofacial and vocal conditional associations are also left-lateralized (See Tables S1 and S3). These findings are generally consistent with existing literature that demonstrated left-dominant recruitment of the ventral prefrontal cortex and the pre-SMA during orofacial and verbal productions (8, 9). By extending this body of work, the present results demonstrate that the acquisition and performance of basic visuo-orofacial and visuo-vocal conditional associations are also left-lateralized. It would be interesting to explore whether this left-lateralization of visuo-orofacial/vocal conditional associative learning and performance is present also in non-human primates. By contrast, activations associated with the processing of vocal feedback during learning in the ventral area 44, as well as the vocal feedback-driven conditional associative learning in the MCC, appeared to be bilateral (Table S3). These results echo previous findings that voice processing recruits the inferior frontal gyrus, bilaterally (10), and that adaptive feedback processing recruits the MCC bilaterally (11).

Supplementary Figure

Fig. S1

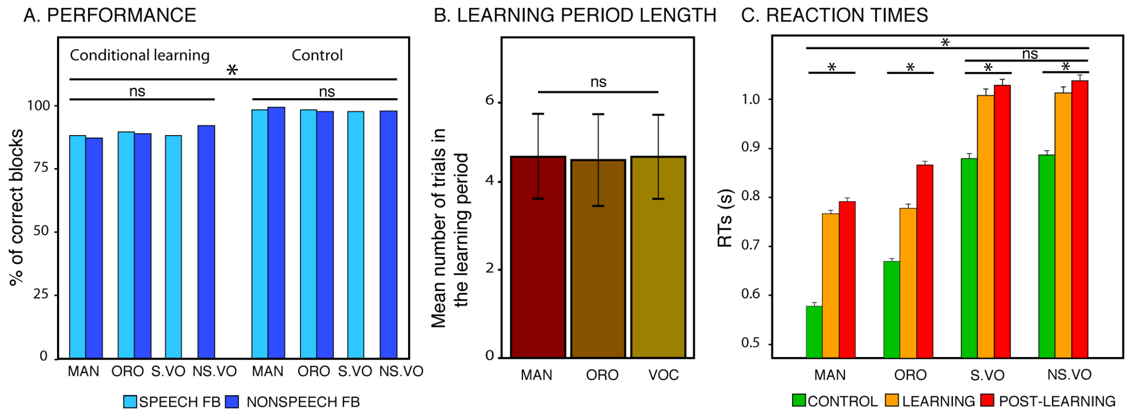

Behavioral data. A. Proportion of blocks successfully completed in the conditional associative learning and control tasks displayed by response types (MAN-manual, ORO-orofacial, S.VO-speech vocal and NS.VO-nonspeech vocal) and feedback types (speech vocal – light blue bars, non-speech vocal – dark blue bars). **B.** Mean number of trials in the learning period in the manual (red), orofacial (orange) and vocal (yellow) response task versions. **C.** Mean reaction times in control (green bars), learning (orange bars) and post-learning (red bars) trials displayed by response types (MAN-manual, ORO-orofacial, S.VO-speech vocal and NS.VO-nonspeech vocal).

Supplementary Tables

Table S1–Increased activations in the posterior lateral frontal cortex observed during manual, orofacial, and vocal conditional selection in the learning and post-learning periods compared to their respective control conditions, i.e. during manual, orofacial, and vocal control selection. Note that in this analysis, the vocal condition includes both speech and nonspeech vocal trials. The x, y, z, coordinates are in MNI stereotaxic space. T-statistics are significant at $p_{corrected}$<0.05.

	**Learning minus Control**				**Post-Learning minus Control**			
	x	*y*	*z*	*t*	*x*	*y*	*z*	*t*
**Manual**								
L. PMd	-26	-4	54	8.79	-30	-12	70	9.50
R. PMd	-	-	-	-	32	-6	68	5.93
L. BA44 (Dorsal)	-54	8	18	8.36	-	-	-	-
L. BA44 (Ventral)	-	-	-	-	-	-	-	-
**Orofacial**								
L. PMd	-26	-2	50	4.19	-30	-8	52	6.48
R. PMd	-	-	-	-	28	-10	52	5.93
L. BA44 (Dorsal)	-54	14	20	6.58	-	-	-	-
L. BA44 (Ventral)	-52	12	10	11.0	-54	10	-4	5.85
**Vocal**								
L. PMd	-	-	-	-	-	-	-	-
L. BA44 (Dorsal)	-50	14	24	6.26	-	-	-	-
L. BA44 (Ventral)	-52	18	6	4.49	-56	8	-2	5.64
L. BA 45	-48	32	-2	4.69	-	-	-	-

Table S2–Increased activations in the medial frontal cortex observed during manual, orofacial, nonspeech vocal, and speech vocal conditional selection in the learning period compared to control in hemispheres with and without a pcgs. X, Y, and Z coordinates correspond to the coordinates of the increased activities in the MNI stereotaxic space. T-statistics are significant at $p_{corrected}$<0.05.

	Hemispheres with a pcgs				Hemispheres without a pcgs			
	x	*y*	*z*	*t*	*X*	*Y*	*z*	*t*
**Manual**								
MCC	|4|	14	52	12.93	|2|	14	50	13.95
MCC	|10|	18	44	8.23	|4|	22	38	10.35
Pre-SMA	-	-	-	-	-	-	-	-
**Orofacial**								
MCC	|2|	14	50	6.92	|6|	14	50	5.81
MCC	|2|	24	38	8.36	|6|	24	42	5.56
Pre-SMA	-	-	-	-	-	-	-	-
**Speech Vocal**								
MCC	|2|	14	54	5.94	|2|	18	50	4.17
MCC	|2|	26	46	6.22	|4|	26	40	4.65
Pre-SMA	|4|	8	68	4.52	|4|	8	72	4.16
**Nonspeech Vocal**								
MCC	|4|	16	50	6.60	|2|	16	50	3.62
MCC	|6|	26	38	3.72	|4|	24	44	3.32
Pre-SMA	-	-	-	-	-	-	-	-

Table S3– Increased activations in the lateral and medial frontal cortex observed during speech and nonspeech vocal feedback analysis in the visuo-manual, visuo-orofacial, visuo-nonspeech vocal, and visuo-speech vocal conditional learning periods compared to control. X, Y, and Z coordinates correspond to the coordinates of the increased activities in the MNI stereotaxic space. T-statistics are significant at $p_{corrected}$<0.05.

	**Speech FB during learning minus control**				**Nonspeech FB during learning minus control**			
	x	*y*	*z*	*t*	*x*	*y*	*z*	*t*
**Manual**								
Left hemisphere								
PMd	-32	-4	66	5.18	-32	-4	64	4.31
MCC	0	14	50	9.93	0	14	50	9.31
MCC	-8	22	46	7.33	-8	22	42	8.35
BA 44 (Ventral)	-50	12	-4	5.20	-58	10	10	3.58
Right hemisphere								
MCC	-	-	-	-	10	20	48	5.84
**Orofacial**								
Left hemisphere								
MCC	-6	20	44	5.30	-8	18	48	5.13
Pre-SMA	-4	4	66	5.12	-	-	-	-
BA 44 (Ventral)	-48	16	8	4.53	-56	14	4	3.34
Right hemisphere								
MCC	4	18	48	4.77	6	22	48	4.80
**Speech Vocal**								
Left hemisphere								
MCC	-6	18	44	5.18	-	-	-	-
Pre-SMA	-6	10	64	4.87	-	-	-	-
BA 44 (Ventral)	-50	12	0	3.35	-	-	-	-
Right hemisphere								
MCC	8	20	48	4.50	-	-	-	-
**Nonspeech Vocal**								
Left hemisphere								
MCC	-	-	-	-	-4	16	50	5.40
Pre-SMA	-	-	-	-	-	-	-	-
BA 44 (Ventral)	-	-	-	-	-52	12	2	4.04
Right hemisphere								
MCC	-	-	-	-	8	20	46	4.15

Table S4–Increased activations in the medial frontal cortex during nonspeech and speech vocal feedback analysis in the visuo-manual, visuo-orofacial, visuo-nonspeech vocal, and visuo-speech vocal conditional learning periods compared to control in hemispheres with and without a pcgs. X, Y, and Z coordinates correspond to the coordinates of the increased activities in the MNI stereotaxic space. T-statistics are significant at $p_{corrected}<0.05$.

	**Hemispheres with a pcgs**				**Hemispheres without a pcgs**			
	x	*y*	*z*	*t*	*x*	*y*	*z*	*t*
**Speech FB / Manual effector**								
MCC	|10|	20	48	7.22	|6|	18	48	8.46
Pre-SMA	-	-	-	-	-	-	-	-
**Nonspeech FB / Manual effector**								
MCC	|2|	16	50	7.20	|8|	22	46	7.48
Pre-SMA	-	-	-	-	-	-	-	-
**Speech FB / Orofacial effector**								
MCC	|6|	18	46	7.93	|2|	18	48	4.22
	|6|	30	34	5.91				
Pre-SMA	|6|	8	62	3.96	|4|	4	66	3.27
**Nonspeech FB / Orofacial effector**								
MCC	|6|	20	46	4.79	|2|	16	50	4.18
Pre-SMA	-	-	-	-	-	-	-	-
**Speech FB / Speech effector**								
MCC	|6|	16	46	7.33	|8|	20	46	4.10
Pre-SMA	|4|	6	64	4.05	|4|	10	64	4.48
**Nonspeech FB / Nonspeech effector**								
MCC	|6|	10	56	7.00				
MCC	|6|	16	48	6.02	|6|	18	46	4.24
Pre-SMA	-	-	-	-	-	-	-	-

Supplementary Movies

Movie S1. Example of visuomotor control task with manual responses and nonspeech vocal feedback.

Movie S2. Example of visuomotor control task with orofacial responses and speech vocal feedback.

Movie S3. Example of conditional learning task with vocal responses and speech vocal feedback.

SI References

1. K. K. Loh, F. Hadj-Bouziane, M. Petrides, E. Procyk, C. Amiez, Rostro-caudal organization of connectivity between cingulate motor areas and lateral frontal regions. *Front Neurosci* **11**, 753 (2018).

2. K. J. Friston, C. D. Frith, R. S. J. Frackowiak, R. Turner, Characterizing dynamic brain responses with fMRI: A multivariate approach. *Neuroimage* **2**, 166–172 (1995).

3. K. J. Friston, C. D. Frith, R. Turner, R. S. J. Frackowiak, Characterizing evoked hemodynamics with fMRI. *Neuroimage* **2**, 157–165 (1995).

4. K. J. Friston, *et al.*, Analysis of fMRI time-series revisited. *Neuroimage* **2**, 45–53 (1995).

5. N. F. Dronkers, A new brain region for coordinating speech articulation. *Nature* **384**, 159–161 (1996).

6. S. Frey, S. Mackey, M. Petrides, Cortico-cortical connections of areas 44 and 45B in the macaque monkey. *Brain Lang* **131**, 36–55 (2014).

7. E. Fedorenko, P. Fillmore, K. Smith, L. Bonilha, J. Fridriksson, The superior precentral gyrus of the insula does not appear to be functionally specialized for articulation. *J Neurophysiol* **113**, 2376–2382 (2015).

8. C. J. Price, A review and synthesis of the first 20 years of PET and fMRI studies of heard speech, spoken language and reading. *Neuroimage* **62**, 816–847 (2012).

9. P. Tremblay, V. L. Gracco, On the selection of words and oral motor responses: Evidence of a response-independent fronto-parietal network. *Cortex* **46**, 15–28 (2010).

10. C. R. Pernet, *et al.*, The human voice areas: Spatial organization and inter-individual variability in temporal and extra-temporal cortices. *Neuroimage* **119**, 164–174 (2015).

11. C. Amiez, F. Hadj-Bouziane, M. Petrides, Response selection versus feedback analysis in conditional visuo-motor learning. *Neuroimage* **59**, 3723–3735 (2012).
